# Opioid-induced short-term consciousness improvement in patients with disorders of consciousness

**DOI:** 10.3389/fnins.2023.1117655

**Published:** 2023-02-03

**Authors:** Qianqian Ge, Yanjun Wang, Yutong Zhuang, Qinghua Li, Ruquan Han, Wenzhi Guo, Jianghong He

**Affiliations:** ^1^Department of Neurosurgery, Beijing Tiantan Hospital, Capital Medical University, Beijing, China; ^2^College of Anesthesiology, Shanxi Medical University, Taiyuan, China; ^3^Department of Neurosurgery, The Second Clinical College of Southern Medical University, Guangzhou, China; ^4^Department of Anesthesiology, Beijing Tiantan Hospital, Capital Medical University, Beijing, China; ^5^Department of Anesthesiology, The Seventh Medical Center of PLA General Hospital, Beijing, China

**Keywords:** disorders of consciousness, vegetative state, minimal conscious state, opioid analgesic, medical treatment

## Abstract

**Introduction:**

Effective treatment to facilitate recovery from prolonged disorders of consciousness is a complex topic for the medical community. In clinical practice, we have found that a subset of patients has a short-term improvement of consciousness after general anesthesia.

**Methods:**

To determine the clinical factors responsible for the consciousness improvement, we enrolled 50 patients with disorders of consciousness who underwent surgery from October 2021 to June 2022. Their states of consciousness were evaluated before surgery, within 48 h after surgery, and 3 months after surgery. Clinical-related factors and intraoperative anesthetic drug doses were collected and compared between patients with and without consciousness improvement. Independent associations between selected factors and postoperative improvement were assessed using multivariate logistical regression analyses.

**Results:**

Postoperative short-term consciousness improvement was found in 44% (22/50) of patients, with significantly increased scores of auditory and visual subscales. Patients with traumatic etiology, a preoperative diagnosis of minimally conscious state, and higher scores in the auditory, visual, and motor subscales were more likely to have postoperative improvement. This short-term increase in consciousness after surgery correlated with patients’ abilities to communicate in the long term. Furthermore, the amount of opioid analgesic used was significantly different between the improved and non-improved groups. Finally, analgesic dose, etiology, and preoperative diagnosis were independently associated with postoperative consciousness improvement.

**Discussion:**

In conclusion, postoperative consciousness improvement is related to the residual consciousness of the patient and can be used to evaluate prognosis. Administration of opioids may be responsible for this short-term improvement in consciousness, providing a potential therapeutic approach for disorders of consciousness.

## 1. Introduction

Critical brain damage, such as traumatic brain injury (TBI), intracerebral hemorrhage (ICH), and anoxic brain injury (ABI), cause disorders of consciousness (DoC). Patients who remain in a state with preserved wakefulness and impaired awareness for >4 weeks are diagnosed with prolonged DoC (Scolding et al., 2021). Many studies have performed further diagnoses in DoC patients to better understand the natural recovery of consciousness (i.e., the ability to respond to external stimuli and to have internal feelings). Depending on the degree of wakefulness and awareness of the patients, DoC is divided into coma (unwakefulness, unawareness), unresponsive wakefulness syndrome/vegetative state (VS) (wakefulness, unawareness), and minimally conscious state (MCS; wakefulness, minimal but definite evidence of awareness) (Thibaut et al., 2019). The diagnosis of MCS is further divided into MCS+ and MCS− by presence or absence of language-related behaviors, respectively, as the functional impairments are different between these two groups of patients (Thibaut et al., 2020).

Despite the significant advances in understanding DoC over the last few decades, there are few valid interventions to promote recovery (Edlow et al., 2021). Several pharmacological treatments are tested for restoring cognitive function after severe brain injuries. Representative classes of drugs are dopaminergic stimulants (e.g., amantadine, methylphenidate, levodopa, bromocriptine, and apomorphine), gamma amino-butyric acid (GABA) receptor stimulants (e.g., zolpidem and baclofen), and antidepressants (e.g., Tricyclic antidepressants) (Pistoia et al., 2010; Ciurleo et al., 2013). However, amantadine is the only pharmacological treatment tested in randomized controlled trials to show benefit in accelerating functional recovery (although it does not improve final outcomes) from acute to subacute traumatic VS and MCS (Giacino et al., 2012). The efficacy of other drugs and treatments is ambiguous, with insufficient evidence and low positive response rates (Fridman and Schiff, 2014). With recent advances in neural arousal circuit research, multiple neuromodulation therapies [e.g., deep brain stimulation and spinal cord stimulation (SCS)], have been used to promote recovery in DoC patients (Xia et al., 2018).

Of interest, during clinical practice with these novel techniques and with more traditional surgical approaches (e.g., cranioplasty and cerebrospinal fluid shunt), we found that a large proportion of patients exhibited a temporary increase in consciousness after general anesthesia, regardless of the type of surgery they received. General anesthesia is the common intervention provided to all patients undergoing surgery. General anesthesia involves a reversible state of unconsciousness, amnesia, analgesia, and dyskinesia induced by a combination of medications, including anesthetics, analgesics, and muscle relaxants (Avidan et al., 2022). The mechanisms by which anesthetic agents induce and maintain the unconscious state, and how consciousness recovers after general anesthesia, are critical issues in neuroscience. Anesthetics can suppress consciousness by inhibiting arousal nuclei in the brainstem and diencephalon (e.g., locus coeruleus and pons reticular formation) or by activating sleep-promoting nuclei (e.g., preventral optic nucleus) (Mashour and Hudetz, 2017). However, opioids, which are the most commonly used analgesics, cause a central excitatory effect during anesthesia recovery (Dzikiti et al., 2016).

The present study examined the hypothesis that the medications used in general anesthesia induce improvements in consciousness in the short-term postoperative period. We retrospectively reviewed 50 DoC patients who underwent surgery and examined the changes in consciousness before and after the operation. Clinically related factors and intraoperative doses of anesthetic drugs were collected and analyzed. The overall aim of this study was to determine the medicines that were responsible for, and that affected the occurrence of short-term consciousness improvement. This information may provide important new information on potential treatments for DoC.

## 2. Materials and methods

### 2.1. Participants

Patients diagnosed with VS or MCS for ≥4 weeks scheduled to undergo surgery in Beijing Tiantan Hospital or the Seventh Medical Center of PLA General Hospital from October 2021 to June 2022 were enrolled in this study. We excluded participants with pre-existing neurological conditions, who took long-acting sedative drugs before the study, and those with liver and kidney failure or serious complications. The overall research protocol was approved by the Ethics Committee of Beijing Tiantan Hospital and the Seventh Medical Center of the Chinese PLA General Hospital. Informed consent was obtained from the legal representatives of the subjects.

### 2.2. Data collection

The demographic and clinical data of all included patients were recorded at baseline. The collected data included age, gender, weight, disease course, and etiology of DoC. Consciousness status was assessed by experienced raters using the revised JFK Coma Recovery Scale (CRS-R) (Kalmar and Giacino, 2005). The scores of six subscales of the CRS-R scale evaluating auditory, visual, motor, oromotor, communication, and arousal functions were recorded. Preoperative CRS-R scores were assessed at least five times within 2 weeks during awake and stable (without complications, including fever, and epilepsy) periods to avoid potential errors caused by fluctuations in responsiveness. The highest score was used to assess each patient’s baseline consciousness level.

The operation methods, the classification of the American Society of Anesthesiologists (Kotake, 2016), and the anesthesia methods were documented. Intraoperative medications, including propofol, sevoflurane, remifentanil, sufentanil, and rocuronium, and their doses were recorded for further analysis. The doses of sufentanil and remifentanil were converted to the morphine dose (Arnold and Weissman, 2003) and added to analyze the total analgesic effect.

After awakening from anesthesia, two experienced physicians screened and recorded each patient’s consciousness state for 48 h. Postoperative consciousness states were also evaluated by the CRS-R scale, and the highest scores were used to describe the patient’s postoperative consciousness level. No treatments or drugs for cortical excitability were used during the first 2 days after surgery. Patients with improved postoperative diagnosis (VS to MCS or MCS− to MCS+) or those who reached the criterion of “localization to sound” or “visual fixation” [reflecting higher-order processing (Weaver et al., 2022)] were classified as the improved group. The remaining patients were classified as the non-improved group. The same assessment was also performed 3 months after surgery.

### 2.3. Statistical analysis

The difference between preoperative and postoperative CRS-R scores and subscores were analyzed with the Wilcoxon signed rank test. To determine the cause of postoperative improvement, explorative data analyses were performed between the improved and non-improved groups. Results are presented as proportions (%), medians and interquartile range, or arithmetic means and standard deviations depending on their scale. Tests for statistical significance were performed with Fisher’s exact test, chi-square test, Wilcoxon–Mann–Whitney *U* test, or two-tailed Student’s *t*-test. Multivariate logistic regression analysis was used to identify independent risk factors for postoperative improvement. A *p*-value < 0.05 was considered statistically significant.

## 3. Results

Fifty DoC patients (18 cases of traumatic brain injury, 16 cases of ICH, and 16 cases of ABI) were enrolled in the present clinical trial between October 2021 and June 2022. The characteristics of all patients are presented in Table 1. All patients suffered from DoC for >1 month. The preoperative diagnosis was determined according to the CRS-R scores [12] and the diagnosis criteria of MCS+ [3]. The median CRS-R score was 7 (range, 4–13). All patients received surgical treatments under general anesthesia, including percutaneous SCS (*n* = 35), SCS (*n* = 11), cranioplasty (*n* = 5), ventriculoperitoneal shunt (*n* = 4), and skin dilator implantation (*n* = 1). Six patients underwent two operations at the same time. The surgical methods were summarized into minimally invasive operations (*n* = 30) and open operations (*n* = 20). There were no significant differences between the surgical techniques, the anesthesia methods, or the anesthetic time.

**TABLE 1:** Baseline demographic and clinical characteristics of the patients.

	Group	Patients (*n* = 50)
Age, years		45.2 ± 15.0
Weight, kg		64.6 ± 8.9
Gender (Male)		30 (60%)
Disease course, months		4.0 [2.0–6.5]
Etiology	TBI	18 (36%)
	ICH	16 (32%)
	ABI	16 (32%)
Diagnosis on admission	VS	29 (58%)
	MCS−	16 (32%)
	MCS+	5 (10%)
CRS-R		7 [6–8]
Severity of surgery	II	4 (8%)
	III	22 (44%)
	IV	24 (48%)
Surgical method	Minimally invasive operation	30 (60%)
	Open operation	20 (40%)
Anesthetic method	Intravenous	14 (28%)
	Inhalation	12 (24%)
	Intravenous-inhalation	24 (48%)
Anesthetic time, minute	–	123 [80–180]

TBI, traumatic brain injury; ICH, intracerebral hemorrhage; ABI, anoxic brain injury; VS, unresponsive wakefulness syndrome/vegetative state; MCS, minimally conscious state; CRS-R, revised JFK coma recovery scale. Data are given as mean ± SD for normal distributed continuous variables, as median [IQR] for abnormal distributed continuous variables, and as count (percentages) for categorical variables.

Postoperative consciousness improvement was found in 22 (44%) patients. The postoperative CRS-R scores of these patients significantly increased (*p* < 0.01) by 1–4 points compared with their preoperative scores (Supplementary Table 1). The scores of the auditory (*p* < 0.01) and visual (*p* = 0.01) subscales increased significantly, with no noticeable change in other subscales. However, this consciousness improvement only lasted 8–48 h after emergence from anesthesia.

### 3.1. Comparisons between the improved and non-improved groups

To analyze the causes and influencing factors of postoperative consciousness improvement, we divided DoC patients into improved and non-improved groups (Table 2). Patients with or without postoperative improvement were significantly different in etiology, preoperative diagnosis, and preoperative CRS-R score (mainly in the auditory, visual, and motor subscales) compared with the non-improved group. Patients with short-term postoperative consciousness improvement also performed better in long-term outcomes compared with the non-improved group. More importantly, patients with short-term postoperative improvement were more likely to regain the ability to communicate (28.6%, two accurate and four intentional) than those without postoperative improvement (7.1%, two intentional). However, the occurrence of postoperative improvement did not predict whether patients would benefit from other treatments (Supplementary Table 2).

**TABLE 2:** Clinical characteristics between the improved and non-improved groups.

	Non-improved	Improved	χ^2^/Z	*P*-value^#^
	*n* = 28 (56%)	*n* = 22 (44%)		
Etiology			6.37^b^	**0.04**
TBI	6 (21.4%)	12 (54.5%)		
ICH	10 (35.7%)	6 (27.3%)		
ABI	12 (42.9%)	4 (18.2%)		
Diagnosis			11.02^b^	**<0.01**
UWS/VS	22 (78.6%)	7 (31.8%)		
MCS−	5 (17.9%)	11 (50%)		
MCS+	1 (3.6%)	4 (18.2%)		
Preoperative CRS-R	6 [5–7]	8 [7–9]	−3.46^a^	**<0.01**
Auditory	1 [1–1]	1 [1–1]	−2.25^a^	**0.02**
Visual	0.5 [0–1]	1 [1–3]	−2.52^a^	**0.01**
Motor	2 [2–2]	3 [2–3]	−3.72^a^	**<0.01**
Oromotor	1 [1–1]	1 [1–1]	0.00^a^	1.00
Communication	0 [0–0]	0 [0–0]	−1.61^a^	0.11
Arousal	2 [2–2]	2 [2–2]	−0.62^a^	0.53
Long-term CRS-R	7 [6–10.5]	10 [8–19]	−2.901^a^	**<0.01**
Auditory	1 [1–2]	1 [1–4]	−1.621^a^	0.11
Visual	1 [0–3]	3 [1–5]	−2.749^a^	**<0.01**
Motor	2 [2–2]	3 [2–5]	−2.637^a^	**<0.01**
Oromotor	1 [1–1]	1 [1–1]	−1.952^a^	0.05
Communication	0 [0–0]	0 [0–1]	−2.045^a^	**0.04**
Arousal	2 [2–2]	2 [2–3]	−2.456^a^	**0.01**
**Intraoperative medication**				
Propofol, mg/kg	1.33 [0–6.22]	3.27 [0.43–10.22]	−1.196^a^	0.23
Sevoflurane,%	1.5 [0.2–2]	1.5 [0–2]	−0.253^a^	0.80
Sufentanil, μg	20 [10–30]	20 [15–22.5]	−0.359^a^	0.72
Remifentanil, μg/kg/min	0.03 [0–0.06]	0.05 [0.05–0.1]	−2.312^a^	**0.02**
Morphine (conversion), mg/kg	0.55 [0.25–1.21]	1.23 [0.61–1.65]	−2.424^a^	**0.02**
Rocuronium, mg	50 [30–50]	45 [30–57.5]	−0.119^a^	0.91

TBI, traumatic brain injury; ICH, intracerebral hemorrhage; ABI, anoxic brain injury; UWS/VS, unresponsive wakefulness syndrome/vegetative state; MCS, minimally conscious state; CRS-R, revised JFK coma recovery scale. Data are given as median [IQR] for abnormal distributed continuous variables, and as count (percentages) for categorical variables. ^a^Wilcoxon–Mann–Whitney test was used to compare groups for continuous variables. ^b^Fisher’s exact test was used to compare groups for categorical variable. ^#^Bold indicates data reaching the threshold of significance (*p* < 0.05).

We also found a significant difference in intraoperative opioid consumption between the two groups. Considering the similar pharmacologic effect of remifentanil and sufentanil, only the converted morphine dose was used in subsequent analyses. By contrast, there were no differences in the other intraoperative medications, surgical methods, anesthetic methods, and anesthetic times (Supplementary Table 2). We further investigated the relationship between the etiology, preoperative diagnosis, and analgesic dose. However, there were no differences in analgesics between patients with different etiologies or diagnoses.

### 3.2. Association with postoperative consciousness improvement

**FIGURE 1:**
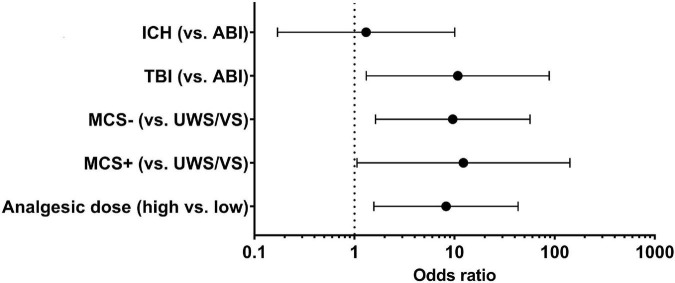
Relative factors of postoperative improvement in DoC patients. In multivariate logistic regression, the etiology (TBI vs. ABI: OR, 10.77; 95% CI, 1.31–88.43; *p* = 0.027), preoperative diagnosis (MCS– vs. VS: OR, 9.6; 95% CI, 1.63–56.73; *p* = 0.013; MCS+ vs. VS: OR, 12.26; 95% CI, 1.06–142.07; *p* = 0.045), and opioid analgesic dose (high vs. low: OR, 8.2; 95% CI, 1.56–43.23; *p* = 0.013) were independently associated with the postoperative improvement in DoC patients. TBI, traumatic brain injury; ICH, intracerebral hemorrhage; ABI, anoxic brain injury; UWS/VS, unresponsive wakefulness syndrome/vegetative state; MCS, minimally conscious state.

Multivariate logistic regression analysis for the endpoint of postoperative consciousness improvement (improved vs. non-improved) was adjusted for etiology (TBI and ICH vs. ABI), preoperative diagnosis (MCS+ and MCS− vs. VS), and analgesic dose (high vs. low). Because of the close relationship between the diagnosis and the CRS-R score (as well as the subscores), we only included the diagnosis in the multivariate logistic regression. To simplify the evaluation, the analgesic doses were divided into high and low groups using the median as the boundary. We found an 8.2-fold higher odds ratio (OR) for postoperative consciousness improvement with a higher analgesic dose [OR, 8.22; 95% confidence interval (95% CI), 1.56–43.23; *p* = 0.013]. Furthermore, etiology (TBI vs. ABI: OR, 10.77; 95% CI, 1.31–88.43; *p* = 0.027) and preoperative diagnosis (MCS− vs. VS: OR, 9.6; 95% CI, 1.63–56.73; *p* = 0.013; MCS+ vs. VS: OR, 12.26; 95% CI, 1.06–142.07; *p* = 0.045) were independently associated with postoperative consciousness improvement (Figure 1).

## 4. Discussion

In the present study, 22 of the 50 patients with DoC experienced a short-term postoperative consciousness improvement. Indeed, postoperative assessment with the CRS-R scale showed a clear and definite improvement in the auditory and visual function of those patients. Patients with traumatic etiology and those with preoperative diagnosis of MCS were more likely to have postoperative improvement. Furthermore, the scores of auditory, visual, and motor subscales were most predictive of postoperative improvement. Finally, this short-term increase in consciousness after surgery correlated with the patients’ abilities to communicate in the long-term. To the best of our knowledge, this is the first clinical report demonstrating the phenomenon of short-term postoperative consciousness improvement in DoC patients. We suggest that opioid analgesics play a critical role in this phenomenon, and that opioids may be a novel therapeutic intervention to promote consciousness and functional recovery in DoC patients.

### 4.1. Relationship between residual consciousness and postoperative consciousness improvement

We found that differences in preoperative diagnosis and etiology of DoC were associated with the incidence of postoperative consciousness improvement. The preoperative CRS-R scores and the auditory, visual, and motor subscores differed between patients in the improved and non-improved groups. Moreover, the patients with short-term consciousness improvement had higher CRS-R scores and the scores of visual, motor, communication, and arousal subscales at 3 months after surgery. Finally, the probability of regaining communication ability was significantly higher in patients in the improved group.

A diagnosis of minimal conscious state was previously defined as reproducible evidence of environmental- or self-awareness (Bender et al., 2015), while a diagnosis of minimal conscious state plus was characterized by the presence of linguistically mediated behaviors (Thibaut et al., 2020). Both states indicated a better-preserved residual consciousness (Laureys et al., 2004). Compared with VS patients, MCS patients demonstrated more widespread brain activation following simple sudatory stimulation and had more robust functional connectivity between the secondary auditory cortex and the temporal and prefrontal auditory-related cortices (Boly et al., 2004). An imageology study also revealed that the dorsomedial body volume of the thalamus was significantly lower in DoC patients, and that this atrophy was more extensive in VS than MCS patients (Fernández-Espejo et al., 2010). Furthermore, significant differences in the N200 and P300 waves of event-related potentials were found between MCS and VS patients (De Salvo et al., 2015). It is generally accepted that traumatic etiology is associated with more favorable outcomes (Ní Lochlainn et al., 2013; Giacino et al., 2018), indicating more complete neural networks in traumatic DoC. The higher rate of postoperative consciousness improvement in patients with traumatic etiology, better preoperative diagnosis, and higher CRS-R scores suggest that short-term improvement may be correlated with better residual consciousness. Given that significant differences were only found in the auditory, visual, and motor subscales in the present study, these subscales may have better predictive power for residual consciousness, which is consistent with our previous findings (He et al., 2014).

We also found that the short-term consciousness improvement was related to the level of consciousness at 3 months after surgery. More importantly, patients in the improved group had significantly higher scores on the communication subscale at 3 months after surgery, and this group had a higher reestablishment rate of communication ability. Although it is unclear whether this long-term improvement was caused by the intraoperative opioid application, the appearance of short-term postoperative improvement may help predict outcomes in DoC patients and guide more aggressive treatments. Communication ability is the most concerning issue for doctors and families of DoC patients, with a great deal of work performed to detect residual consciousness and potential communication abilities (Owen and Coleman, 2008; Gui et al., 2020). Complex methods, including structural (Sattin et al., 2021) and functional (He et al., 2015; Aubinet et al., 2020) magnetic resonance imaging, electroencephalography (Bai et al., 2021; Porcaro et al., 2022), and brain–computer interfaces (Müller-Putz et al., 2013), are used to evaluate residual consciousness and prognosis. However, changes in patient performance can affect the assessment and prognosis accuracy in DoC (Murovec et al., 2020). Our findings raise the possibility that a short-term administration of opioids (or remifentanil) may improve patients’ performance and assist in assessing residual consciousness.

### 4.2. Role of opioids analgesics in postoperative consciousness improvement

We found no significant differences in postoperative consciousness improvement for any intraoperative medications except remifentanil. When combining the doses of remifentanil and sufentanil by converting to the morphine dose, the difference was even more marked. The lack of a difference with sufentanil treatment between the two groups may be related to use of a similar dose to that for induction drugs by the anesthesiologists. However, in subsequent multivariate regression analysis, the dose of opioid analgesics was an independent factor affecting short-term postoperative improvement.

Both remifentanil and sufentanil are opioid agonists, which produce an analgesic effect by activating the μ-opioid receptor (MOR) (Ziesenitz et al., 2018). Opioid receptors are widely distributed in the central nervous system. MORs located in the dorsal horn of the spinal cord, a major center of pain information processing (Braz et al., 2014), are essential for both the analgesic effects and the sensory input potentiation of opioids (Sun et al., 2019). The opioid system also potently modulates the mesolimbic circuitry, limbic circuitry, cortical and hippocampal circuitry, and various brain regions underlying motivation, fear responses, and cognitive functions (Puryear et al., 2020). MORs are predominantly expressed on GABAergic inhibitory interneurons and exert a potent disinhibitory effect on excitatory neurons (Nam et al., 2021).

There is a paradoxical hyperalgesia response in patients receiving opioids for pain control, whereby some patients become more sensitive to painful stimuli (hyperalgesia) and have a painful reaction to innocuous stimuli (allodynia)—this is termed opioid-induced hyperalgesia (OIH) (Velayudhan et al., 2013). The mechanism of OIH is not fully understood but is generally considered related to sensitization of the pronociceptive pathway caused by peripheral and central neuroplastic changes (Lee et al., 2011). OIH has been widely reported after the perioperative use of opioid analgesia (Colvin et al., 2019). A meta-analysis of OIH after surgery comparing 1,494 patients from 27 studies found that a higher intraoperative opioid dose (mainly remifentanil) was correlated with higher postoperative pain intensity and morphine use (Fletcher and Martinez, 2014). A biphasic time-dependent effect of fentanyl was also reported (Célèrier et al., 2000), with a nociceptive threshold increase lasting 2–5 h after fentanyl injection, followed by a sustained descending nociceptive threshold for up to 5 days.

The degree of hyperalgesia is dose-dependent. The occurrence of postoperative consciousness improvement was only found in patients receiving higher doses of opioids. Moreover, the time points of the consciousness improvement (within 48 h post-operation) and OIH were similar. The pattern between the onset of hyperalgesia and opioid analgesia use was similar to that between the short-term postoperative consciousness improvement and the use of opioids found in the present study, suggesting a relationship between OIH and consciousness improvement. However, we found no difference between minimally invasive and open surgery, indicating that the incision pain was not responsible for the consciousness improvement. Sensitization of the pronociceptive pathway is achieved *via* sensitization of primary afferent neurons and second-order neurons, an increased concentration of excitatory neurotransmitters (through enhanced production and release and diminished reuptake), and activation of descending facilitation of the rostral ventromedial medulla (Lee et al., 2011). These mechanisms ultimately lead to enhanced sensory afferent signals, which cause algesia in conscious patients. However, in patients with DoC, the same physiological responses to opioids strengthen the external environmental stimulation, enhancing the signal input to the ascending reticular activating system. This activation maintains cortical neurons in a state of facilitation and excitation, leading to a better clinical manifestation in DoC patients. Although this side effect of opioids is unwanted by anesthesiologists, it has the potential to become a therapy to promote recovery of consciousness in DoC patients. Further studies assessing the mechanisms of hyperalgesia in DoC are required.

Another potential mechanism underlying the postoperative improvement in consciousness with opioids involves enhanced dopamine release caused by the disinhibitory effect of activated GABAergic neurons. Opioids can activate MORs in the reward circuitry of the brain, which suppresses inhibitory neurotransmission in the ventral tegmental area and reduces the inhibitory postsynaptic event frequency of GABAergic interneurons, further increasing the release of dopamine into the striatum and prefrontal cortex (Colvin et al., 2019). This opioid-mediated disinhibition of dopaminergic neurons in the ventral tegmental area and substantia nigra pars compacta is hypothesized to cause the arousing and rewarding effects of opioids (Steidl et al., 2017). Recently, the occurrence of forebrain dysfunction in DoC was found to be caused by death and disconnection of neurons as well as “circuit-level” functional disturbances, which could be modulated to promote the recovery of consciousness (Schiff, 2010). The “mesocircuit” hypothesis suggests that normal anterior forebrain function depends on activation of thalamocortical projections in the central thalamus, which is inhibited by globus pallidus internal tonic signals during DoC (Schiff, 2008). High levels of dopaminergic activity maintain striatal firing rates, which inhibits the tonic signals of the globus pallidus and further activates the central thalamus (Grillner et al., 2005). The only validated treatment for DoC, amantadine (Giacino et al., 2012), is an indirect dopamine agonist. Increased striatal D2 dopamine-receptor availability and prefrontal cortical metabolism were found after amantadine treatment (Kraus et al., 2005; Schnakers et al., 2008). The same pattern of dopaminergic neuron activation is also found after opioid administration.

There are a number of other potential mechanisms involved in the beneficial effects of opioids. The latest clinical guidelines recommend evaluating and treating pain in patients with DoC (Giacino et al., 2018). However, as a subjective experience, it is difficult to recognize pain in DoC patients when no self-report is available. Nevertheless, recent advances in neuroimaging techniques have provided the capacity to perceive pain in DoC patients (Schnakers and Zasler, 2015). The high prevalence of spasticity in DoC patients and the positive correlation between the level of spastic muscle overactivity and pain (Thibaut et al., 2015) suggest that a large proportion of these patients suffer pain. Persistent pain may affect patients’ responses to the external environment, and pain relief *via* administration of analgesics may improve the clinical manifestations in DoC patients. Recent studies have shown that the pain-related brain circuit is incomplete in VS patients, with less evidence of painful conscious experiences. By contrast, MCS patients have sufficient cortical integration to process nociceptive stimuli, and the patterns of brain activation to painful stimulation in MCS patients were similar to those in healthy controls (Schnakers and Zasler, 2007). These differences in pain experience between VS and MCS patients may partly explain the different rates of postoperative improvement between these two groups.

### 4.3. Limitations

This preliminary study included a small sample of patients. Further studies with more patients and standardized within-group differences are required. Furthermore, we only assessed the level of consciousness during the first two postoperative days because additional treatments introduced more distractors on the following days. It should also be noted that because of the lack of responsiveness in DoC patients, a wide range of the anesthetic agents were applied to our patients. Standardized anesthesia protocols should be stipulated in future studies.

## 5. Conclusion

Short-term consciousness improvement is related to patients’ residual consciousness and can aid estimation of long-term prognosis. Opioid analgesics may cause short-term improvements in consciousness *via* enhanced sensory afferent signals caused by (i) opioid-induced peripheral and central neuroplastic changes, (ii) increased striatal dopamine release caused by disinhibition of opioid-related GABAergic neuron activation, and (iii) relief of persistent pain in DoC patients. These findings suggest that opioids may be useful for determining prognosis and promoting recovery in DoC patients. However, further clinical and experimental studies are required to understand the utility of opioids in DoC patients, including opioid-related consciousness improvement.

## Data availability statement

The raw data supporting the conclusions of this article will be made available by the authors, without undue reservation.

## Ethics statement

The studies involving human participants were reviewed and approved by Ethics Committee of Beijing Tiantan Hospital and the Seventh Medical Center of the Chinese PLA General Hospital. Written informed consent to participate in this study was provided by the participants or their legal guardian/next of kin.

## Author contributions

JH, QG, and YW contributed to conception and design of the study. QG, YW, YZ, and QL organized the database. QG and YW performed the statistical analysis. QG wrote the first draft of the manuscript. YW wrote sections of the manuscript. All authors contributed to manuscript revision, read, and approved the submitted version.
