# Supplementary material for: Opioid-induced short-term consciousness improvement in patients with disorders of consciousness
Source: Front Neurosci. 2023 Feb 3;17:1117655. doi: 10.3389/fnins.2023.1117655 (PMC9936155; doi:10.3389/fnins.2023.1117655)
Supplement: Supplementary file 1 [file Table_1.docx]

Supplementary Material

Opioid-induced Short-term Consciousness Improvement in Patients with Disorders of Consciousness

# Supplementary Table 1

| **Table S1** CRS-R scores of the patients with postoperative improvement | | | | |
| --- | --- | --- | --- | --- |
|  | **Preoperative** | **Postoperative** | **Z** | **p** |
| **CRS-R; median [[IQR]** | 8 [7-9.25] | 9.5 [8.75-12] | -4.242 | **＜0.001** |
| Auditory | 1 [1-1.25] | 2 [1-2.25] | -3.207 | **0.001** |
| Visual | 1 [1-3] | 2.5 [1-3] | -2.565 | **0.010** |
| Motor | 3 [2-3] | 3 [2-3] | -1.633 | 0.102 |
| Oromotor | 1 [1-1] | 1 [1-1] | -1 | 0.317 |
| Communication | 0 [0-0] | 0 [0-0] | 0 | 1.000 |
| Arousal | 2 [2-2] | 2 [2-2] | 0 | 1.000 |

CRS-R: revised JFK Coma Recovery Scale
Bold indicates data reaching the threshold of significance (P < 0.05)
