# Supplementary material for: Opioid-induced short-term consciousness improvement in patients with disorders of consciousness
Source: Front Neurosci. 2023 Feb 3;17:1117655. doi: 10.3389/fnins.2023.1117655 (PMC9936155; doi:10.3389/fnins.2023.1117655)
Supplement: Supplementary file 2 [file Table_2.docx]

Supplementary Material

Opioid-induced Short-term Consciousness Improvement in Patients with Disorders of Consciousness

Supplementary Table 2

**Table S2** Other clinical characteristics between the improved and non-improved groups

|  | **Non-improvement** | **Improvement** | **t/χ^2^/Z** | **p** |
| --- | --- | --- | --- | --- |
|  | n=28 | n=22 |  |  |
| **Age, years; mean (SD)** | 44.14 (15.54) | 46.55 (14.46) | -0.56^a^ | 0.579 |
| **Weight, kg; mean (SD)** | 66.68 (9.20) | 61.86 (7.89) | 1.95^a^ | 0.057 |
| **Gender: Male** | 17 (60.7%) | 13 (59.1%) | 0.01 ^c^ | 0.907 |
| **Disease course, months; median [IQR]** | 4.5 [2.25-8] | 4 [1.5-6] | -0.80 ^b^ | 0.424 |
| **Severity of surgery** |  |  | 0.62 ^c^ | 0.830 |
| II | 3 (10.7%) | 1 (4.5%) |  |  |
| III | 12 (42.9%) | 10 (45.5%) |  |  |
| IV | 13 (46.4%) | 11 (50%) |  |  |
| **Surgical method** |  |  | 0.22 ^c^ | 0.642 |
| Minimally invasive operation | 16 (57.1%) | 14 (63.6%) |  |  |
| Open operation | 12 (42.9%) | 8 (36.4%) |  |  |
| **Anesthetic method** |  |  | 0.79 ^c^ | 0.673 |
| Intravenous | 7 (25%) | 7 (31.8%) |  |  |
| Inhalation | 8 (28.6%) | 4 (18.2%) |  |  |
| Intravenous-inhalation | 13 (46.4%) | 11 (50%) |  |  |
| **Anesthetic time, minute; median [IQR]** | 138 [66.25-180] | 120 [92.5-180] | -0.44 ^b^ | 0.660 |
| **Long-term improvement** |  |  | 0.41 ^c^ | 0.522 |
| Non-improvement | 14 (50%) | 9 (40.9%) |  |  |
| Improvement | 14 (50%) | 13 (59.1%) |  |  |

Data are given as mean±SD for normal distributed continuous variables, as median [IQR] for abnormal distributed continuous variables, and as count (percentages) for categorical variables;
^a^ Two-tailed Student’s t-test was used for normally distributed continuous variables;
^b^ Wilcoxon–Mann–Whitney test was used for abnormal distributed continuous variables;
^c^ Fisher’s exact test was used for categorical variable.
